# The Metabolism of Polysaccharide from *Atractylodes macrocephala* Koidz and Its Effect on Intestinal Microflora

**DOI:** 10.1155/2014/926381

**Published:** 2014-11-19

**Authors:** Ruijun Wang, Guisheng Zhou, Mengyue Wang, Ying Peng, Xiaobo Li

**Affiliations:** School of Pharmacy, Shanghai Jiao Tong University, Shanghai 200240, China

## Abstract

An active polysaccharide from the rhizome of *Atractylodes macrocephala* Koidz (PAM) was identified to improve and adjust disordered intestinal flora. High-performance gel permeation chromatography (HPGPC) and gas chromatography-mass spectrometry (GC-MS) were employed to identify the components of PAM as rhamnose, glucose, mannose, xylose, and galactose at a ratio of 0.03 : 0.25 : 0.15 : 0.41 : 0.15. PAM metabolized in gastrointestinal tract when incubated with artificial gastric and intestinal juices. Anaerobic incubation of PAM on intestinal flora confirmed that PAM promoted the ability of intestinal bacteria to digest reducing sugar. Based on the Shannon index and similarity coefficient index of enterobacterial repetitive intergenic consensus-PCR (ERIC-PCR) fingerprinting of the total intestinal bacteria DNA, we concluded that PAM can significantly improve disordered intestinal flora and may be used as an oral adjuvant to regulate intestinal flora.

## 1. Introduction

Recent studies reported that the rising incidences of bowel disease were caused by gut microbes [1, 2]. These microbes were also associated with many epidemics chronic illnesses such as diabetes mellitus [3], inflammatory bowel disease [4], and obesity [5]. The gut microbes can be affected by many factors [6–9] including diet, body state, and environment, among which dietary was the major factor. Currently, it remains unclear how gut bacteria respond to active components from diets or functional foods. Studies on the relationship between intestinal flora and active components are warranted.

Reports indicated that polysaccharides can impact on the colonic microbiota [10–12], but its mechanistic interaction with intestinal flora remains unknown. Further studies on such interactions would be useful to understand the physiological benefits of polysaccharide and provide instructions for proper therapeutic uses.

Polysaccharides are important constituent of many well-known functional herbs also in traditional Chinese medicine (TCM) [13], such as* Atractylodes* [14–16], ginseng [17], and* Poria cocos* [18]. At present, the rhizome of* Atractylodes macrocephala* Koidz, the most common functional food, is known to regulate the gut microbes and is widely used in the treatment of chronic intestinal disease [15].

In this study, a novel polysaccharide (PAM) was isolated from the rhizome of* A. macrocephala* with improved metabolic effect on intestinal microflora and stability in artificial gastric/intestinal juice. By using anaerobic incubation and ERIC-PCR fingerprinting methods, the effect of PAM on intestinal bacteria* in vitro* and* in vivo* was evaluated. This study provided novel understanding on physiological and therapeutic benefits of polysaccharides on intestinal flora.

## 2. Experimental Section

### 2.1. Materials, Reagents, and Preparation of CAE

Dried rhizome of* Atractylodes macrocephala* Koidz (batch number HX20081102) and the folia of* Cassia angustifolia* Vahl (batch number HX20081103) were bought from Shanghai Huayu Pharmaceutical co., Ltd. (Shanghai, China). Their botanical origins were identified by the corresponding author, and voucher specimens were deposited at the School of Pharmacy, Shanghai Jiao Tong University. DEAE-52 was bought from Whatman (Maidstone, Britain) and Sephacryl S-100 gel was supplied by Amersham (Uppsala, Sweden). Monosaccharaides were purchased from Sigma (St. Louis, USA). Primers ERIC 1R (5′-ATGTAAGCTCCTGGGGATTCAC-3′) and ERIC 2R (5′-AAGTAA GTGACTGGGGTGAGCG-3′) were obtained from Sangon Biotech Co., Ltd. (Shanghai, China). All reagents were of analytical grade.

The dry powder of the folia of* C. angustifolia *(senna) was extracted (CAE) thrice with boiling water (1 : 10) for 120 min (40 min each time). The three filtrates were merged and evaporated by rotary evaporation under vacuum at 60°C. Finally, the concentration of extracts was set at 1.0 g/mL.

### 2.2. Animals

Male SD rats (190–200 g and 5 weeks old) were purchased from Shanghai Slack Laboratory Animal co., Ltd. (Shanghai, China). All procedures related to the animals and their care conformed to the internationally accepted principles as found in the Guidelines for Keeping Experimental Animals issued by the Government of China.

### 2.3. Extraction, Isolation, and Purification of Polysaccharides

The dried powder of the rhizome of* A. macrocephala* (200 g) was extracted thrice with 2000 mL boiling water for 3 h (1 h each time). The aqueous extracts were filtrated, combined, and concentrated to about 200 mL with rotary evaporators. To precipitate the crude polysaccharide, 3-fold volume of pure ethanol was added to the extracts at 4°C overnight. The resulting precipitate was collected by centrifugation, washed with pure ethanol (400 mL) repeatedly, and dried under nitrogen. 20 g dry precipitate was obtained, reconstituted in 1000 mL pure water, and deproteinized using 3-fold volume of Sevag reagent (*n*-butanol/chloroform, v/v = 1 : 4). Finally, 900 mL supernatant was collected, concentrated to 100 mL, and lyophilized to 13.8 g crude polysaccharides.

To purify the isolated polysaccharides, 1 g crude polysaccharides were dissolved, filtered through 0.45 *μ*m filters, and applied to a DEAE-52 cellulose chromatography column (26 mm × 60 cm). The crude polysaccharides were fractionated and eluted with 1000 mL distilled water at a flow rate of 1 mL/min. The water fractions obtained were determined according to the total carbohydrate content by the phenol-sulfuric acid method using glucose as the standard. The water elutes with polysaccharides were combined, concentrated to 100 mL, and divided into ten 10 mL aliquots. Each aliquots was fractionated by size-exclusion chromatography on a Sephacryl S-100 gel chromatography column (24 mm × 45 cm) eluted with 400 mL pure water at a flow rate of 0.5 mL/min each time. The phenol-sulfuric acid method was also employed in this process. The water eluting fraction was collected and concentrated to 50 mL and lyophilized to 0.05 g white purified polysaccharide (PAM).

### 2.4. Structure Analysis of PAM

The molecular weight of PAM was determined by high-performance gel permeation chromatography (HPGPC) [19]. The monosaccharide composition of PAM was determined by GC-MS [20]. The IR spectrum of PAM was determined using a Fourier transform IR spectrophotometer (FT-IR) (NEXUS-870, Nicolet Instrument Co., USA) [21]. Microstructure was determined by atomic force microscopy [22].

### 2.5. The Stability of PAM in Artificial Gastric/Intestinal Juice

The artificial gastric and intestinal juices were prepared referenced to Chinese Pharmacopoeia [23]. Briefly, 40 mL artificial gastric or intestinal juice was added to 40 mL PAM solutions (1 mg/mL in pure water) and incubated in water bath at 37°C. During incubation, 0.8 mL mixed solution was taken at 0, 1, 2, 3, 4, 5, 6, 12, and 24 h, respectively. The concentration of polysaccharide was determined in 0.4 mL solution using phenol-sulfuric acid method [24], and the concentration of reducing sugar was tested in 0.4 mL solution using DNS method [25]. The metabolic rate of PAM was calculated by the following formula:

(1) $$\begin{array}{c} M\%=\frac{\left[I\%-\left(T-R\%\right)\right]}{I\%}\times 100\%, \end{array}$$

where *M* is metabolic rate; *I* is initial concentration of polysaccharide; *T* is total concentration of polysaccharide; *R* is concentration of reducing sugar.

### 2.6. The Effect of PAM on Human/Rat Intestinal Bacteria* In Vitro*

The human intestinal bacteria juice was prepared as previously reported [26]. Briefly, PAM was blended with human/rat intestinal flora liquid in anaerobic incubation and incubated at 37°C. During incubation, 4 mL mixture incubated was taken at 0, 2, 4, 6, 8, 10, 12, and 24 h, respectively. The digestion of reducing sugar was determined using DNS method. The calculation formula of reducing sugar digestion rate was given as follows:

(2) $$\begin{array}{c} R\%=\frac{\left(I\%-L\%\right)}{I\%}\times 100\%, \end{array}$$

where *R* is reducing sugar digestion rate; *I* is initial level of reducing sugar; *L* is remaining level of reducing sugar.

### 2.7. The Effect of PAM on the Intestinal Flora* In Vivo*

After one week of acclimatization, the rats were randomly divided into 4 groups (with 8 rats in each group), namely, control (healthy), model (untreated), and high- (0.105 g/kg) and low-dose (0.035 g/kg) PAM treated groups. The rats of model and high- and low-dose PAM groups were intragastrically given CAE 10 g/kg (10 g crude herbs per 1 kg rat body weight) twice a day for the first 10 d to induce the disordered model of intestinal flora. From the eleventh day, the high- and low-dose PAM groups were treated with PAM solution in dose of 0.105 g/kg and 0.035 g/kg one time each day for 10 d, respectively. The control and model groups were intragastrically given the same volume of distilled water.

Three or four pieces of fecal pellets (about 1.0 g) per rat were directly collected from the anus into sterile plastic tubes and stored at −20°C. Procedures were repeated after 8 h. Fecal pellets were collected at the 1st, 10th, and 17th days of the animal experiment.

The protocol to extract total DNA of intestinal bacteria from feces and method to perform ERIC-PCR were described previously [27]. Statistical analysis was carried out using the Shannon diversity index and similarity coefficients (similarity degree of the two classification units). Shannon's diversity index was the value to describe the community diversity of intestinal flora, and it was calculated as follows:

(3) $$\begin{array}{c} H^{\prime }=\sum \left(pi\right)\left(\text{lo}{\text{g}}_{2}p-i\right), \end{array}$$

where *p* represents the proportion of a phylotype relative to the sum of all phylotypes of intestinal flora. The similarity coefficient was calculated as

(4) $$\begin{array}{c} C_{s}\left(\%\right)=\frac{\left(2\;\times \;j\right)}{\left(a+b\right)}\times 100\%, \end{array}$$

where “*a*” is the number of total bands in the ERIC-PCR pattern for one sample, “*b*” is the number for the other, and “*j*” is the number of the common bands shared by both samples [27].

## 3. Results and Discussion

### 3.1. Structure Characterization Analysis of PAM

**Figure 1:**
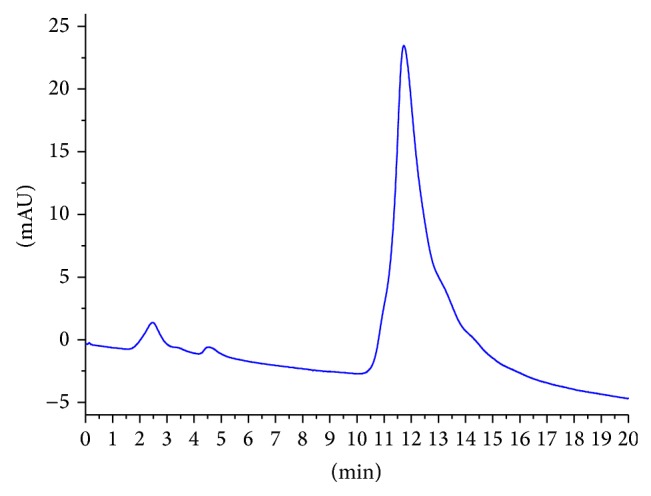
High-performance gel permeation chromatography of PAM.

**Figure 2:**
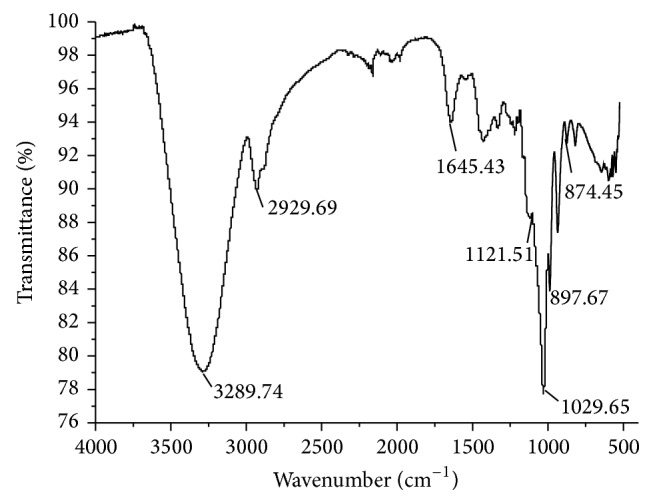
FTIR of PAM.

**Figure 3:**
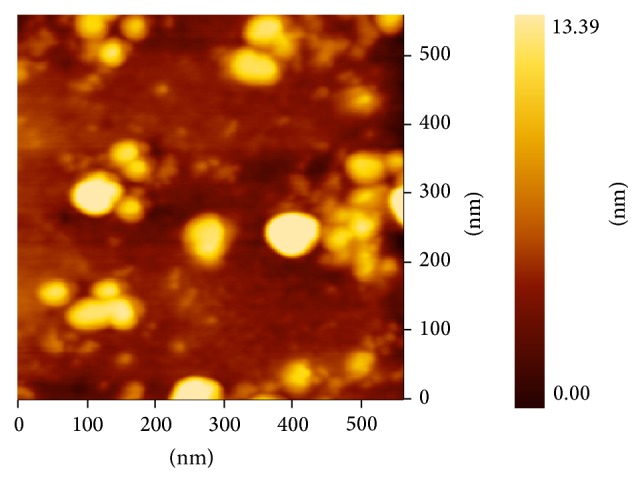
Atomic force microscopy images of PAM.

**Figure 4:**
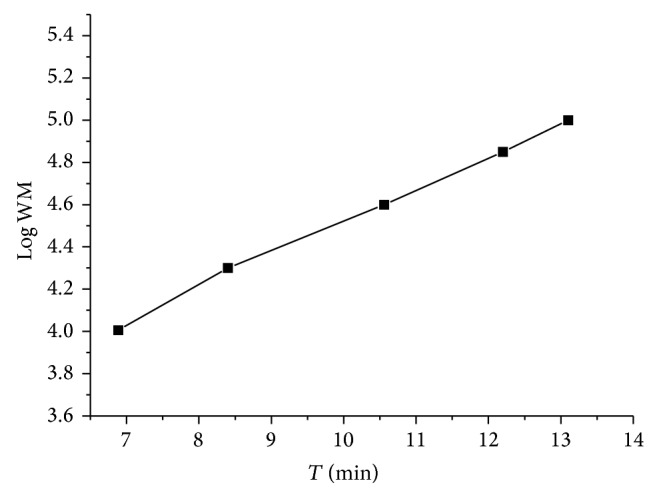
Standard curve of the molecular weight of PAM.

**Figure 5:**
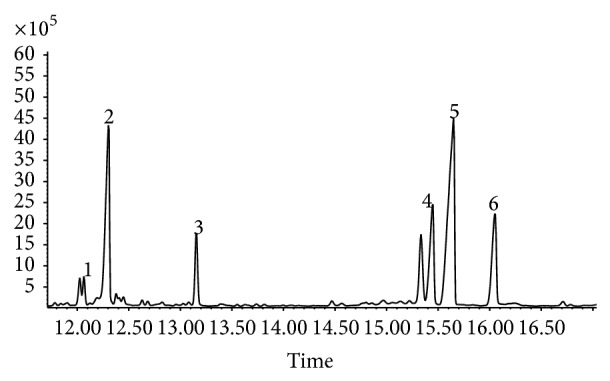
The PAM derivative GC-MS chromatogram. 1: Rhamnose; 2: xylose; 3: inositol; 4: glucose; 5: mannose; 6: galactose.

PAM was shown as a single and symmetrical peak in HPGPC (Figure 1). From the chromatography, PAM was a homogeneous polysaccharide. The FT-IR spectrum of PAM (Figure 2) revealed a major broad stretching peak at 3289.74 cm^−1^ for the typical hydroxyl group and a weak band at 2929.69 cm^−1^ for the C–H stretching vibration [21]. The absorbance at 1651.98 cm^−1^ indicated the presence of carbonyl group. The main absorptions of C–O stretching 1121.51 cm^−1^ suggested that the characteristics of sugar structures were pyranose configuration. The band at 874.45 cm^−1^ indicated the existence of mannose in PAM. The band at 897.46 cm^−1^ was the characteristic peak of *β*-configuration in PAM. The PAM image taken from atomic force microscope was shown in Figure 3 and the mode of PAM molecular chain was winding coil and spherical distribution and multiple nodular polysaccharide combined with each other by intermolecular interactions. The diameter of the molecular size was 42.2 nm under the magnification of the image display. The molecular weight of PAM was 28773 Da as calculated by drawing standard curve (Figure 4). The monosaccharide composition (Figure 5) of PAM was rhamnose, glucose, mannose, xylose, and galactose, and the mole ratio was 0.03 : 0.25 : 0.15 : 0.41 : 0.15. The result of monosaccharide composition indicated that the percentage of xylose was very high in PAM. Previous studies reported that human intestinal* Bifidobacterium* can be activated and proliferated by xylose [28] with enhanced benefits for human health. Consumption of xylose can improve the microbial environment of the human body. So, it provided the basis for the following research about PAM function.

### 3.2. The Stability of PAM in Artificial Gastric/Intestinal Juice

**Figure 6:**
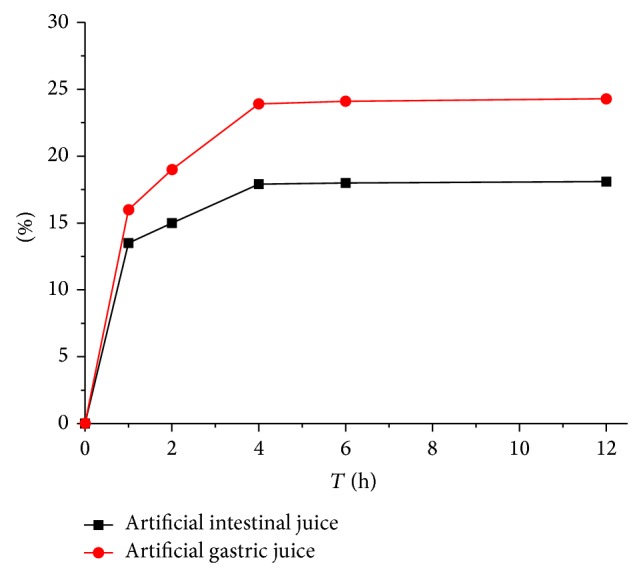
Metabolism of PAM in artificial intestinal juice/gastric juice.

Figure 6 shows the changes of PAM metabolism in artificial gastric/intestinal juice in a 12-hour time window. The concentration of reducing sugar produced increased significantly in the first 1 h, followed by a slow increase until equilibrium. At 12 h, the concentration of reducing sugar in the artificial gastric and intestinal juice was 24.3% and 18.1%, respectively. The results indicated that PAM was metabolized in gastrointestinal tract. Moreover, the majority part of PAM was digested in the intestinal juice.

### 3.3. The* In Vitro* Activity of PAM on Intestinal Flora

**Figure 7:**
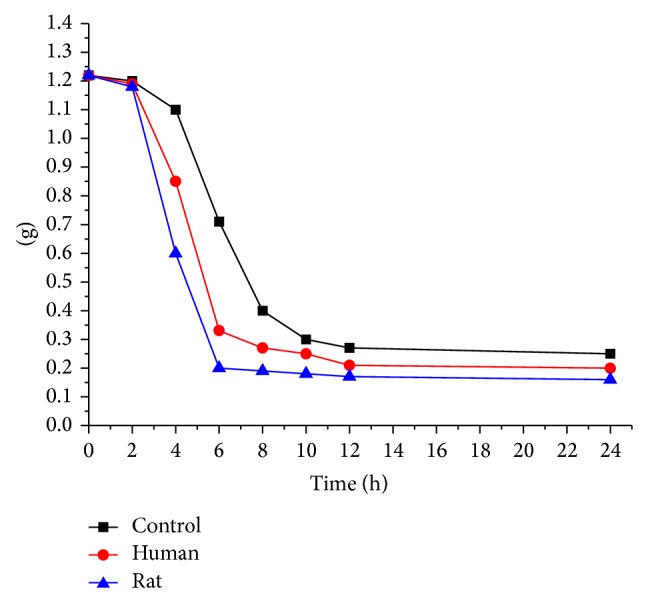
The effect of PAM on human/rat intestinal flora to digest reducing sugar.

When PAM was added and anaerobically incubated with human/rat intestinal bacterial mixture from feces, it was found that the consumption rate of reducing sugar was increased.  Figure 7 shows how the content of reducing sugar changed in human, rat, and control. The changes of PAM content might be divided into three main periods: slow reduction (0~2 h), sharp reduction (2~6 h), and smooth reduction (6~24 h). From 2 to 6 h, the consumption rates of reducing sugar decreased significantly and almost linearly (1.2–0.2 g and 1.2–0.3 g in human and rat, resp.). In general, the human intestinal flora was similar to rat on the consumption rate of reducing sugar incubated with PAM 77.9% and 76.9%, respectively. Therefore, PAM might activate and accelerate the growth of intestinal flora, which provided clues for the further study of PAM* in vivo*.

### 3.4. The* In Vivo* Metabolic Activity of PAM on Intestinal Flora

**Figure 8:**
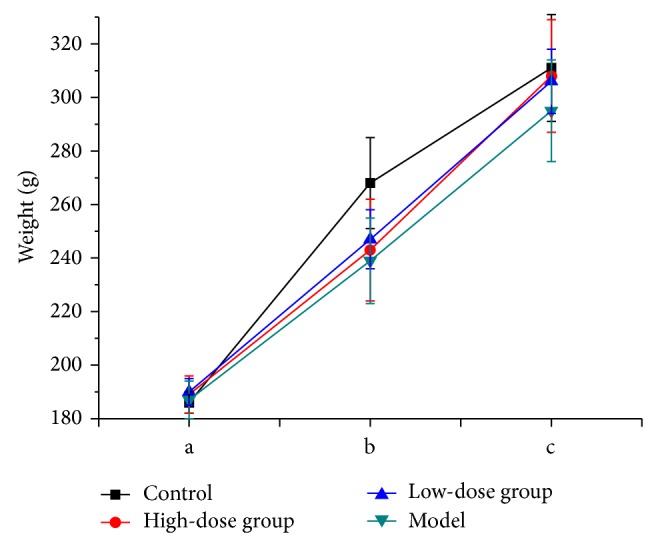
The weight of rats at the periods of modeled and treated (a: before administration; b: after administration; c: after treatment).

A model animal of intestinal flora disordered was established by oral administration of CAE [27]. The characteristic symptom of watery stools was obviously observed in the model rats, and the other symptoms were also observed in this study, such as humped back, narrow eyes, listlessness, inappetence, and weight loss. When treated by PAM intragastrically, the characteristic symptom of watery stools was significantly improved. Their vigor was increased and their weights were obviously recovered in groups receiving high and low dose of PAM (Figure 8). Compared to the untreated group, the symptoms in all treated groups reduced more efficiently, but there were no significant differences between groups.

**Figure 9:**
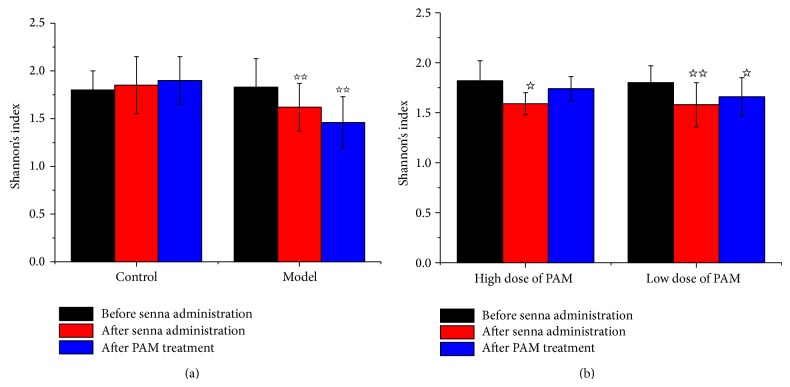
Shannon's diversity index of ERIC-PCR fingerprinting of the disordered intestinal flora model of rats before and after treatment. (a) Shannon's diversity index of control group and model groups. (b) Shannon's diversity index of high dose and low dose of PAM groups. (*P* ^☆^ < 0.05,* P* ^☆☆^ < 0.01, compared with before senna folium administration.)

ERIC-PCR profiles of intestinal flora DNA from feces were performed on 32 rats (Figure 9). In healthy condition (control group), the Shannon diversity index (*H*′) of 32 rats was 1.81 ± 0.02, while model group (untreated) dropped to 1.6 ± 0.02 (*P* < 0.05) with a decline of 20%. It was shown that the ecological balance of intestinal flora in rats was destroyed and flora species were declined. When the model rats were administrated with PAM, the *H*′ was increased to 1.74 ± 0.04 (*P* < 0.05, compared to model group) in high-dose group. However, Shannon's diversity index of low dose of PAM group was maintained at a constant level, and there was no significant difference compared with model group. A certain content of PAM might be very important to increase the diversity index of model group.

**Figure 10:**
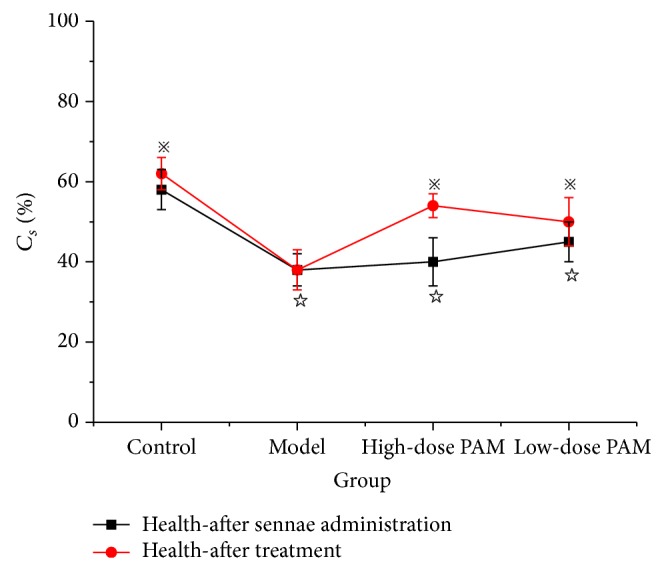
Similarity coefficient index (*C*_*s*_%) of ERIC-PCR fingerprintings of rat intestinal flora before and after PAM treatment. Control: Group 1, received distilled water in both inducement and treatment phases. Model: Group 2, received senna administration but distilled water during treatment; High-dose PAM and Low-dose PAM: Group 3 and Group 4, received high and low doses of PAM repectively during treatment. (*P* ^☆^ < 0.05, compared with the control;* P* ^*※*^ < 0.05 compared with the model.)

As shown in Figure 10, Sorenson index (*C*_*s*_) was used to compare ERIC-PCR fingerprinting of intestinal flora of different condition rats. Before and after being treated by CAE,* C*_*s*_ of models (groups: model, high-dose PAM, and low-dose PAM) ranged from 38% to 45%. Comparing with the control group (58%),* C*_*s*_ of model group showed a significant decrease (*P* < 0.05). For high and low dose of PAM groups, intestinal flora in each treatment group presented a certain degree of recovery, especially high-dose group (54%, *P* < 0.05, compared to the model group). Both high and low dose of PAM could improve similarity coefficients of ERIC-PCR fingerprinting, which indicated that PAM could significantly improve the structure of intestinal flora. Furthermore, it was reported that consumption of PAM showed an improvement of intestinal function [29]. Studies on the change of intestinal flora structure by PAM treatment can provide reference to understand the underlying mechanisms of polysaccharides on intestinal flora.

## 4. Conclusions 

In this paper, an active polysaccharide of PAM was isolated from the rhizome of* A. macrocephala*, and its structure was also identified. ERIC-PCR profile analyses were successfully employed to investigate the effect of PAM on the intestinal flora. Our results demonstrated that PAM could improve and adjust the disordered intestinal flora, suggesting that PAM may have the potential as an oral adjuvant for disordered intestinal flora. This study also provided a novel conception and evidences for future investigation of the metabolic effect of polysaccharides on intestinal flora and their therapeutic effects.
